# Clinical Usefulness of Measuring Red Blood Cell Distribution Width in Patients with Hepatitis B

**DOI:** 10.1371/journal.pone.0037644

**Published:** 2012-05-23

**Authors:** YuFeng Lou, ManYi Wang, WeiLin Mao

**Affiliations:** Department of Clinical Laboratory, First Affiliated Hospital, Zhejiang University School of Medicine, Hangzhou, Zhejiang Province, People’s Republic of China; Drexel University College of Medicine, United States of America

## Abstract

**Background:**

Red blood cell distribution width (RDW), an automated measure of red blood cell size heterogeneity (*e.g.*, anisocytosis) that is largely overlooked, is a newly recognized risk marker in patients with cardiovascular diseases, but its role in persistent viral infection has not been well-defined. The present study was designed to investigate the association between RDW values and different disease states in hepatitis B virus (HBV)-infected patients. In addition, we analyzed whether RDW is associated with mortality in the HBV-infected patients.

**Methodology/Principal Findings:**

One hundred and twenty-three patients, including 16 with acute hepatitis B (AHB), 61 with chronic hepatitis B (CHB), and 46 with chronic severe hepatitis B (CSHB), and 48 healthy controls were enrolled. In all subjects, a blood sample was collected at admission to examine liver function, renal function, international normalized ratio and routine hematological testing. All patients were followed up for at least 4 months. A total of 10 clinical chemistry, hematology, and biochemical variables were analyzed for possible association with outcomes by using Cox proportional hazards and multiple regression models. RDW values at admission in patients with CSHB (18.30±3.11%, *P*<0.001), CHB (16.37±2.43%, *P*<0.001) and AHB (14.38±1.72%, *P*<0.05) were significantly higher than those in healthy controls (13.03±1.33%). Increased RDW values were clinically associated with severe liver disease and increased 3-month mortality rate. Multivariate analysis demonstrated that RDW values and the model for end-stage liver disease score were independent predictors for mortality (both *P*<0.001).

**Conclusion:**

RDW values are significantly increased in patients with hepatitis B and associated with its severity. Moreover, RDW values are an independent predicting factor for the 3-month mortality rate in patients with hepatitis B.

## Introduction

Red cell distribution width (RDW) is an automated measure of the heterogeneity of red blood cell (RBC) sizes (*e.g.* anisocytosis) and routinely performed as part of a complete blood cell counts [1]–[3]. RDW is used in the differential diagnosis of anemia [4]. Recently, a series of studies have demonstrated that RDW can serve as a novel, independent predictor of prognosis in patients with cardiovascular diseases (*e.g.* heart failure [5]–[7], stable coronary diseases [8], acute myocardial infarction [9], strokes [10], and pulmonary hypertension [11]). Elevated RDW values were also shown to be associated with increased risk of mortality in the general population [12]–[14]. However, to our knowledge, the role of RDW values in persistent viral infection has not been well-defined. More importantly, whether RDW values are associated with different disease states of hepatitis B virus (HBV) infection such as acute hepatitis B (AHB), chronic hepatitis B (CHB) and chronic severe hepatitis B (CSHB) remains unknown. The present study was designed to investigate the association between RDW values and different disease states in HBV-infected patients. In addition, we analyzed whether RDW is associated with mortality in the HBV-infected patients.

## Materials and Methods

### Subjects

Adult HBV-infected patients admitted to the First Affiliated Hospital of Zhejiang University School of Medicine and diagnosed with AHB, CHB or CSHB were consecutively recruited between August 1, 2010 and August 1, 2011. In the present study, whereas there were no exclusions for age/sex, patients who received any anti-HBV agents or steroids 6 months before admission were excluded. Patients with a concurrent infection of HCV, hepatitis D virus, hepatitis G virus, and/or human immunodeficiency virus and any autoimmune liver disease were also excluded. During the same time period, age- and sex-matched healthy individuals were recruited as controls at a patient/control ratio of 3∶1.

Blood samples were collected from all HBV-infected patients within 24 hours after admission, and blood samples were taken from 48 healthy individuals at the time of recruitment. After discharge, all patients were followed up monthly by phone conversation, and every three months by patient’s visit to the hospital. Blood samples were collected at each visit for laboratory tests including the detection of HBsAg. Neither the technician who performed the laboratory tests nor the investigator who followed up the patients knew the diagnosis of the patients.

The study was approved by the Ethics Committee of the First Affiliated Hospital of Zhejiang University School of Medicine, and written informed consent for participation was obtained from each study participant.

### Clinical Diagnosis

The diagnostic criteria for AHB, CHB and CSHB were in accordance with the 2000 Xi’an Viral Hepatitis Management Guidelines recommended by the Chinese Society of Infectious Diseases and Parasitology, and the Chinese Society of Hepatology, of the Chinese Medical Association [15]. Briefly, viral hepatitis B is classified into three major clinical types, namely AHB, CHB and CSHB. AHB is defined as when hepatitis B surface Ag (HBsAg)-negative conversion occurs within 6 months after the initial onset of symptoms due to HBV infection. CHB is defined as when a HBV carrier requires a clinical course of hepatitis B infection for more than 6 months and may have exhibited symptoms or signs of hepatitis and abnormal hepatic function, or with histological changes. CSHB is defined as when there is a history of CHB or liver cirrhosis with serum HBsAg positivity of more than 6 months and a serum total bilirubin level of more than 10 times the normal level (*i.e.* 171 µmol/L), with at least one of the following five liver failure indexes: prothrombin activity of less than 40%, hepatic encephalopathy, ascites, progressive reduction in liver size, and hepatorenal syndrome. In addition, the diagnosis of liver cirrhosis was made on the basis of clinical (*e.g.* physical stigmata of cirrhosis), biochemical (*e.g.* decreased serum albumin and increased serum globulin levels), and ultrasonographic or computed tomography (*e.g.* nodular liver surface, coarsened echogenicity of liver parenchyma, enlarged spleen, and/or ascites) findings [16], [17].

### Laboratory Methods

RDW, hemoglobin level, and mean corpuscular volume (MCV) were determined using the XE-2100 automated hematology analyzer (Sysmex Corp, Kobe, Japan), as one part of a complete blood cell count. The normal reference range for RDW in the laboratory of our hospital is 11.6% –15.0%. Serum creatinine, serum albumin, total protein, total bilirubin and alanine transaminase levels were measured using the Hitachi 704 Analyzer (Boehringer Mannheim Diagnostics), and the international normalized ratio (INR) was generated by the Sysmex CA1500 full-automatic analyzer (Sysmex Corp, Hyogo, Japan). At baseline, demographic and clinical characteristics, including the model for end-stage liver disease (MELD) score (with higher scores indicating more severe illness), were collected.

### MELD Score

Liver disease severity at admission was evaluated *via* the MELD score, which uses the patient’s serum bilirubin and creatinine levels and the INR for prothrombin time to predict survival. The MELD score was calculated using the web site calculator (http://www.mayoclinic.org/gi-rst/mayomodel7.html).

### Statistical Analysis

All continuous variables were expressed as mean value ± standard deviation (SD), and categorical data as percentages. We used SPSS version 15.0 (SPSS, Inc., Chicago, IL) to perform statistical procedures. The Kruskal-Wallis H test and Mann-Whitney nonparametric U test were used for comparison between groups. Categorical data were evaluated by the χ^2^-test or Fisher’s exact test, as appropriate. A multivariable stepwise logistic regression test was used to evaluate independent clinical parameters predicting mortality. The receiver operating characteristic (ROC) curve was obtained and area under the curve (AUC) was calculated to identify the best RDW to predict mortality in patients with HBV infection. A value of *P*<0.05 was considered statistically significant.

## Results

### Increased RDW Values in HBV-infected Patients

A total of 123 HBV-infected patients, 16 with AHB, 61 with CHB, and 46 with CSHB, as well as 48 healthy individuals, were recruited during the study period (Table 1).

**Table 1:** Clinical characteristics of studied subjects.

	AHB(n = 16)	CHB(n = 61)	CSHB(n = 46)	Healthy controls(n = 48)
Age (year) *	39.8±13.0	44.5±12.1	49.9±13.5	43.3±10.9
Gender (male/female)	11/5	46/15	36/10	36/12
Total bilirubin (µmol/L) *	107±54	65±13	304±150	12±5
Alanine aminotransferase (U/L) *	1027±818	111±146	123±118	24±11
International normalized ratio*	1.39±0.44	1.13±0.23	2.23±1.18	0.91±0.21
HBsAg positive	16	61	46	0
HBeAg positive	7	61	20	0
HBcAb IgM positive	16	0	0	0
HBV DNA positive	10	61	22	0
Mortality	0	6	35	0

* Data are expressed as mean ± standard deviation.

AHB, Acute hepatitis B; CHB, Chronic hepatitis B; CSHB, Chronic severe hepatitis B; HBsAg, Hepatitis B surface Antigen; HBeAg, Hepatitis B e Antigen; HBcAb, Hepatitis B core Antibody; HBV, Hepatitis B virus.

**Figure 1:**
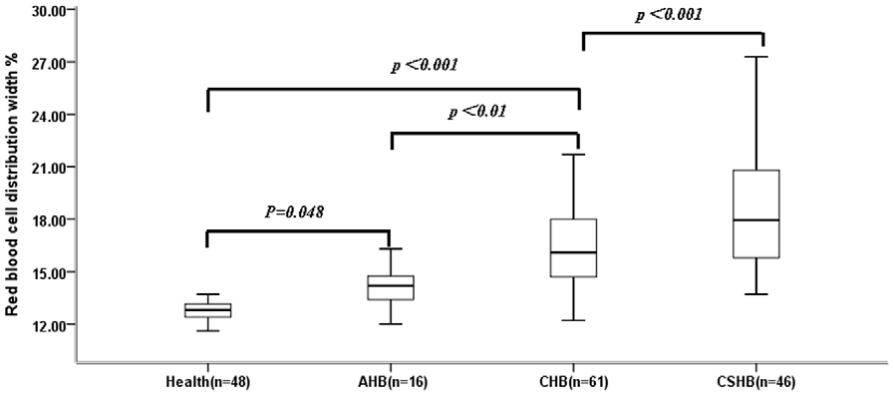
The association between red cell distribution width (RDW) values and different disease states in HBV-infected patients. Data are expressed as box plots, in which the horizontal lines illustrate the 25th, 50th, and 75th percentiles of the values of RDW. The vertical lines represent the 5th and 95th percentiles. AHB, Acute hepatitis B; CHB, Chronic hepatitis B; CSHB, Chronic severe hepatitis B.

The RDW values of HBV-infected patients at admission ranged from 12.0% to 27.3%. The values in patients with CSHB patients (18.30±3.11%, *P*<0.001), CHB patients (16.37±2.43%, *P*<0.001) and AHB patients (14.38±1.72%, *P*<0.05) were significantly higher than those in healthy controls (13.03±1.33%). Moreover, CSHB patients had higher RDW values than CHB and AHB patients (both *P*<0.001), and CHB patients had higher RDW values than AHB patients (*P*<0.05) (Figure 1).

### Baseline Characteristics and Baseline Factors Related with RDW

Patients were divided into three groups based on their RDW values: group A (RDW ≤15.0%), group B (>15.0%, but <20.0%) and group C (≥20.0%). Differences in clinical and laboratory characteristics among the three groups of RDW are listed in Table 2. Patients with higher values of RDW tended to be older, were more likely to have severe liver disease, had lower levels of hemoglobin, total protein, and serum albumin, and had higher INR and total bilirubin. The serum creatinine, gender and MCV were not significantly different among the three groups.

**Table 2:** Clinical and laboratory characteristics among patients with different red blood cell distribution width (RDW) values at admission.

	Group A(RDW≤15.0%, n = 47)	Group B(15.0<RDW<20.0%, n = 53)	Group C(RDW≥20.0%, n = 23)	*P*
RDW (%)*	13.95±0.94	17.05±1.41	21.70±1.59	<0.001
Age (year) *	41.8±13.2	48.7±12.1	54.7±11.5	0.003
Gender (male/female)	39/8	44/9	16/7	0.077
MCV (fL) *	92.8±5.2	92.5±8.2	89.8±13.2	0.087
Hemoglobin (g/dL) *	133.8±16.3	115.6±18.6	94.4±21.8	<0.001
Total Protein (g/L) *	65.7±6.2	61.2±6.1	59.0±5.9	<0.001
Albumin (g/L) *	38.8±6.2	33.7±5.0	32.9±2.9	<0.001
Alanine aminotransferase (U/L) *	433.1±658.1	128.9±143.1	66.8±31.5	0.003
International normalized ratio *	1.33±0.41	1.53±0.47	1.99±0.63	<0.001
Creatinine(mmol/L) *	63.4±14.9	65.7±17.8	68.9±33.5	0.544
Total bilirubin (µmol/L) *	105.7±115.3	164.7±140.5	250.1±198.7	0.001
MELD score *	10.7±4.2	15.1±4.7	19.8±4.4	<0.001
Hepatic cirrhosis (yes/no)	5/42	20/33	19/4	<0.001
Ascites (yes/no)	2/45	12/41	18/5	<0.001
mortality (yes/no)	6/41	19/34	16/7	<0.01

* Data are expressed as mean±standard deviation.

MCV, mean corpuscular volume; MELD score, model for end-stage liver disease score; RDW, red cell distribution width.

**Figure 2:**
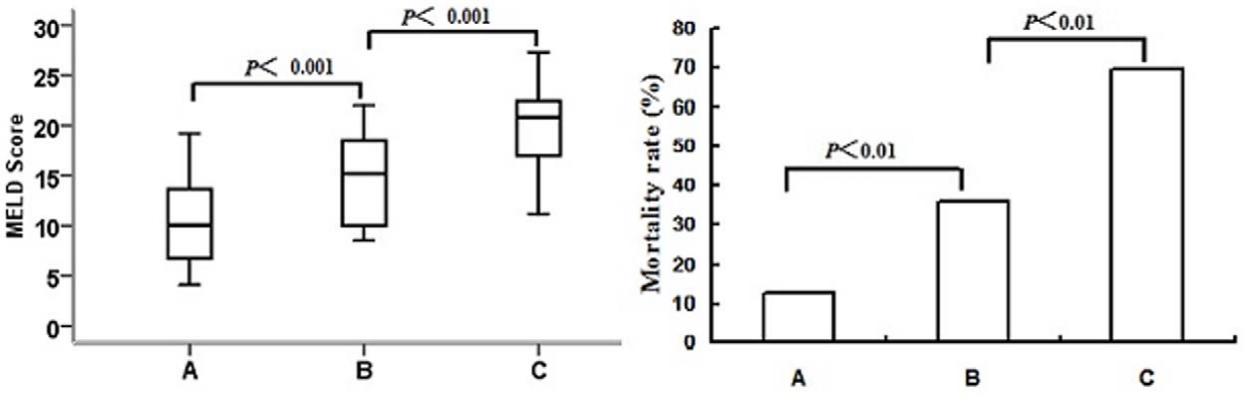
Comparisons of the Model for End-stage liver disease (MELD) score (left) and mortality rate (right) among patients with different red cell distribution width (RDW) values. Patients were divided into three groups based on serum RDW values: group A (≤15.0%), B (>15.0%, but <20.0%) and C (≥20.0%).

The MELD score in groups A, B, and C were 10.7±4.2, 15.1±4.7 and 19.8±4.4, respectively. There was a stepwise increase in MELD scores with increasing values of RDW (*P*<0.001 between groups A and B, and between groups B and C) (Figure 2, left).

### Association of RDW with 3-month Mortality in HBV-infected Patients

The median follow-up period was 72 days (range, 21–128 days). During the follow-up, 41 patients died, none of whom were patients with AHB, six were with CHB and 35 were with CSHB.

There was a significantly increase in the 3-month mortality rate following increasing RDW values, with 12.7% in group A, 35.8% in group B and 69.6% in group C (*P*<0.01 between groups A and B, and between groups B and C) (Figure 2, right).

**Figure 3:**
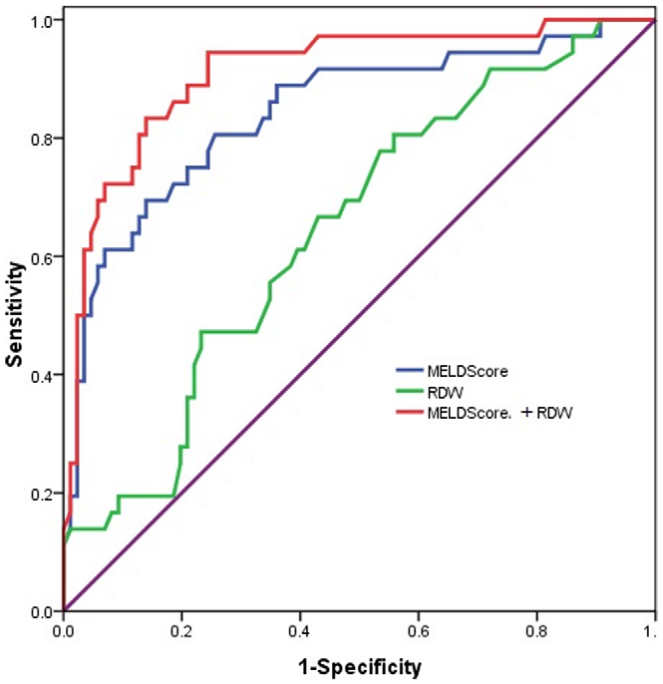
Receiver operating characteristic (ROC) curve analysis for prediction of mortality by red cell distribution width (RDW) values (green line), Model for End-stage Liver Disease (MELD) scores (blue line) and their combination (red line) at admission.

To evaluate the values for RDW and MELD score to predict mortality, ROC curves were drawn (Figure 3). The AUCs were calculated as 0.847±0.034 for the MELD score and 0.664±0.049 for RDW (both *P*<0.001). When RDW and MELD were combined, the AUC was 0.905±0.019 (*P*<0.001). The multivariate logistic regression analysis showed that only the RDW values and MELD score were independent factors predicting mortality rate (Table 3).

**Table 3:** Independent predictors of mortality by multivariate logistic regression analysis.

Predictor	Odds ratio	95% CI	*P*
RDW (%)	1.973	1.290–2.173	0.027
MELD Score	2.474	1.980–3.047	0.005

Variables included in the analysis were sex, age, hemoglobin, RDW, mean corpuscular volume, MELD score, serum albumin, total protein, international normalized ratio, and total bilirubin.

MELD, model for end-stage liver disease; RDW, red cell distribution width.

## Discussion

RDW reflects the variability in circulating RBC size. It is based on the width of the RBC volume distribution curve, with larger values indicating greater variability [18]. RDW is elevated when there is increased red cell destruction, or, more commonly, ineffective red cell production. RDW may represent a nutritional deficiency (*e.g.* iron, vitamin B12, or folic acid), bone marrow depression, or chronic inflammation [19]–[21]. These conditions are often present in patients with liver disease, correlate with the severity of the disease, and are associated with a worse prognosis [22]. In our study, patients with hepatitis B had significantly higher RDW values compared with healthy subjects and CSHB patients had the highest RDW values among the patients. Thus, we speculate that this difference is an important factor that influences the disease progression, and may present an important marker for patients with HBV infection.

The most significant finding from our study is that increasing RDW values can serve as an independent predictor of mortality in HBV-infected patients. Over the past decade, the MELD score has emerged as the most widely used model for organ allocation in liver transplantation. This model, which includes variables related to both liver and renal function, was implemented in the USA in 2002 and is currently being used in many countries to classify patients with cirrhosis awaiting transplantation according to the severity of their liver disease [23]. Our previous study reported that the MELD score was related to the prognosis of the patients with HBV-related acute-on-chronic liver failure [24]. In the present study, we reported that RDW can be used for predicting HBV-infected patients mortality, although the prediction power of RDW was relatively lower (AUC = 0.664±0.049 *P<*0.001) than that of MELD score (AUC = 0.847±0.034, *P<*0.001). Moreover, combining RDW with the MELD score further added to prediction power of predicting mortality (AUC = 0.905±0.019, *P*<0.001). This is more relevant to patients with CSHB. The present study included 16 patients with AHB, 61 with CHB and 46 with CSHB. It is well-known that AHB patients are relatively less commonly observed in the clinical setting because 90–95% of adult patients generally have a spontaneously self-limited acute hepatitis without obvious manifestation and often develop the convalescence period through a short-term acute phase before they see doctors. Thus, a 3-month mortality rate is relatively low in patients with AHB. Indeed, in the present study, we found that none of patients with AHB died, whereas six patients with CHB and 35 patients with CSHB died before the 3-month follow-up period.

The mechanisms underlying the association between RDW and severity of hepatitis and its role in predicting mortality in HBV-infected patients are unclear. Recently, in a large unselected cohort of patients, RDW showed a strong and graded association with inflammatory markers, which was independent of ferritin, age, sex, and other haematological variables [25]. Inflammation might contribute to increased RDW values not only by impairing iron metabolism but also by inhibiting the production of or response to erythropoietin or by shortening RBC survival [26], [27]. Indeed, a number of studies have shown that proinflammatory cytokines suppress erythropoietin gene expression, inhibit proliferation of erythroid progenitor cells, down-regulate erythropoietin receptor expression, and reduce erythrocyte life-span [27]. Inflammation in a HBV-infected liver is proven to be mediated by cytokines that play a pivotal role in the pathogenesis of chronic HBV infection [28], [29]. A number of cytokines are released from macrophages or monocytes in response to stimulation by endotoxins, and these cytokines affect disease status [30]. We hypothesize that the association of RDW with an increased mortality risk may, in part, be due to an effect of inflammation on anisocytosis and risk. Moreover, as shown in the present study, we also found an association between RDW and the severity of the liver disease, and patients with higher values of RDW tended to be older, had lower levels of hemoglobin, total protein, and serum albumin, and had higher INR and total bilirubin. Meanwhile, a typical anemia with a high RDW was found in liver cirrhosis principally in relation to the disease severity [31]. Several studies of the RBC mass and plasma volume in cirrhosis have shown that there is an expanded plasma volume in the presence of portal hypertension, leading to low hematcrit [32], and that RBC survival is also reduced in cirrhotic patients, especially those with anemia, although blood loss may partly contribute to the reduced RBC survival [33]. Hemolysis appears to be the major cause of anemia in advanced cirrhosis, in which an enlarged spleen sequesters and destroys blood cells efficiently, leading to macro-normoblastic bone marrow [34].

It should be mentioned that, in the present study, AHB, CHB and CSHB were diagnosed in accordance with the 2000 Xi’an Viral Hepatitis Management Guidelines recommended by the Chinese Society of Infectious Diseases and Parasitology, and the Chinese Society of Hepatology of the Chinese Medical Association [15]. In China, the term ‘CSHB’ is usually used for the fatal form of chronic hepatitis, which resembles the term ‘liver failure caused by chronic hepatitis B” in Western countries. Although the term is not uniformly used globally, this chronic liver condition often demonstrates serious clinical courses with fatal consequences. Acute attacks may occur in some patients with CHB. Moreover, the disease may develop into liver failure (*i.e.* CSHB) due to various factors, such as HBV mutations [35], [36], coinfection with other hepatotropic viruses [37]–[42], over-work, alcohol overdose [43], long-term corticosteroid treatment [44], and bacterial infections [45], during the long course of the disease. Although the application of newly developed drugs with an artificial liver support system is effective in some cases, the mortality rate of CSHB may still reach 80%–100% in patients with stage III–IV hepatic encephalopathy [46]. For these patients, orthotopic liver transplantation [47] might be the last option. Therefore, identification of novel predicting biomarkers is critical in therapeutical management for CSHB.

A few limitations warrant consideration. First, we did not investigate the causes of elevated RDW values, such as iron or vitamin B12 deficiency, which could confound the association between RDW values and adverse outcome. Second, this was a single-center study and thus our relatively small sample size may have posed a limitation to this study. Therefore, our findings need to be confirmed in multi-center and prospectively designed studies. Finally, RDW values were not dynamically observed, and thus, whether RDW values are stepwise elevated when patient’s condition is progressively deteriorated remains unclear.

In conclusion, RDW values are significantly increased in patients with hepatitis B and associated with the severity. Moreover, RDW values are an independent predicting factor for the 3-month mortality rate in patients with hepatitis B. Because RDW values are easily attainable at no additional cost to the routine complete blood cell counts and is highly reproducible, it may serve as an important biomarker. The strength of RDW’s association with mortality risk that we and others have observed compares favorably with established risk factors. It is unknown, however, whether the risk associated with RDW is modifiable or if RDW itself is modified by current therapies that alter prognosis.
